# Modelling Highly Biodiverse Areas in Brazil

**DOI:** 10.1038/s41598-019-42881-9

**Published:** 2019-04-23

**Authors:** Ubirajara Oliveira, Britaldo Silveira Soares-Filho, Adalberto J. Santos, Adriano Pereira Paglia, Antonio D. Brescovit, Claudio J. B. de Carvalho, Daniel Paiva Silva, Daniella T. Rezende, Felipe Sá Fortes Leite, João Aguiar Nogueira Batista, João Paulo Peixoto Pena Barbosa, João Renato Stehmann, John S. Ascher, Marcelo F. Vasconcelos, Paulo De Marco, Peter Löwenberg-Neto, Viviane Gianluppi Ferro

**Affiliations:** 1 Centro de Sensoriamento Remoto, Instituto de Geociências, Universidade Federal de Minas Gerais – UFMG, Av. Antonio Carlos 6627, CEP 31270-901 Belo Horizonte, MG Brazil; 2 Departamento de Zoologia, Instituto de Ciências Biológicas, Universidade Federal de Minas Gerais – UFMG, Av. Antonio Carlos 6627, CEP 31270-901 Belo Horizonte, MG Brazil; 3 Departamento de Biologia Geral, Instituto de Ciências Biológicas, Universidade Federal de Minas Gerais – UFMG, Belo Horizonte, MG Brazil; 4 Laboratório Especial de Coleções Zoológicas, Instituto Butantan, São Paulo, SP Brazil; 5 Departamento de Zoologia, Universidade Federal do Paraná, Curitiba, Paraná Brazil; 6 Instituto Federal Goiano – IFGoiano, Departamento de Biologia, Urutaí – Goiás, Brazil; 7 Sección Palentología de Vertebrados Museo Argentino de Ciencias Naturales “Bernardino Rivadavia” Avenida Angel Gallardo 470, C1405DJR Buenos, Aires, Argentina; 8 Laboratório Sagarana, Instituto de Ciências Biológicas e da Saúde, Universidade Federal de Viçosa – UFV, Campus Florestal, Florestal, MG Brazil; 9 Departamento de Botânica, Instituto de Ciências Biológicas, Universidade Federal de Minas Gerais – UFMG, Belo Horizonte, MG Brazil; 10 Department of Biological Sciences, National University of Singapore, Singapore, Singapore; 11 Instituto Prístino, Rua Santa Maria Goretti, 86, Barreiro, CEP 30642-020 Belo Horizonte, MG Brazil; 12 Departamento de Ecologia, Instituto de Ciências Biológicas, Universidade Federal de Goiás, Goiânia, Goiás Brazil; 13 Universidade Federal da Integração Latino-Americana, Foz do Iguaçu, PR Brazil

**Keywords:** Biodiversity, Conservation biology

## Abstract

Traditional conservation techniques for mapping highly biodiverse areas assume there to be satisfactory knowledge about the geographic distribution of biodiversity. There are, however, large gaps in biological sampling and hence knowledge shortfalls. This problem is even more pronounced in the tropics. Indeed, the use of only a few taxonomic groups or environmental surrogates for modelling biodiversity is not viable in mega-diverse countries, such as Brazil. To overcome these limitations, we developed a comprehensive spatial model that includes phylogenetic information and other several biodiversity dimensions aimed at mapping areas with high relevance for biodiversity conservation. Our model applies a genetic algorithm tool for identifying the smallest possible region within a unique biota that contains the most number of species and phylogenetic diversity, as well as the highest endemicity and phylogenetic endemism. The model successfully pinpoints small highly biodiverse areas alongside regions with knowledge shortfalls where further sampling should be conducted. Our results suggest that conservation strategies should consider several taxonomic groups, the multiple dimensions of biodiversity, and associated sampling uncertainties.

## Introduction

Biodiversity conservation strategies focus on protecting most species at the least cost^1^. Usually, the ratio between the number of species and lineages, especially endemic, since the latter are more susceptible to extinction^1,2^, and size of the area prioritized is a good indicator of the effectiveness of any given strategy approach. Regions that present higher α and β diversity as well as more species endemism and phylogenetic diversity and endemism are considered as highly biodiverse and thus relevant for conservation^1,3^. Mapping areas of biological relevance along with their respective degrees of knowledge is, therefore, a first step towards an effective conservation planning^3–5^.

Traditional approaches for identifying relevant areas assume adequate, representative, and uniform biodiversity sampling^1,6,7^. Yet systematic, all-encompassing sampling is not the case for most regions of the planet^4,5,8^. The incomplete sampling of species occurrences entails “biological knowledge shortfall”. Main biological knowledge shortfalls, *i.e*. insufficient information on a particular biological dimension, include the Linnaean (number of species), Wallacean (species geographic distribution), Hutchinsonian (species niche), and Darwinian (evolutionary relationships between species)^9^. These knowledge shortfalls are more conspicuous in the tropics, home to the largest biological diversity^6,9^, and hence may directly impair conservation strategies designed for these regions. Indeed, conservation strategies designed in more intensely sampled regions, such as Europe and North America, do not usually address knowledge shortfalls and as a result may produce unsatisfactory results when applied to tropical countries such as Brazil^7^.

Previous studies attempted to solve this problem by using taxonomic surrogates, which are usually groups of birds or mammals whose geographic distribution is presumably better known^10,11^. However, the knowledge about the geographic distribution of these groups may also be incomplete^5^. In addition, there is little evidence to support the hypothesis that these groups’ diversity and distribution adequately represents other organisms. Another drawback to this approach is that it does not deal with the knowledge shortfalls inherent to biodiversity modelling^1^. One alternative is to use environmental surrogates, such as biophysical features, as predictors of biotic variation across space in species distribution models (SDM), *e.g*. Maxent^12^. Though useful, this strategy does not overcome the problem that sampling is usually insufficient to cover all the spatial variation of correlated biophysical features. In addition, the use of biomes or ecoregions as biogeographical units of analysis in conservation studies assumes that these regions have unique species composition, which is far from the rule^1,11^. Lastly, few of previous studies for modelling biodiversity conservation priorities have assessed the effectiveness of their approaches, nor have they included phylogenetic information^11,13–15^.

To overcome these limitations, we developed and evaluated a spatially-explicit model, named OCEUB (Optimizing Combined Evidences in Unique Biota). Our model applies a genetic algorithm tool for identifying within regions of unique biota the smallest possible areas that contain the most number of species and phylogenetic diversity, as well as the highest endemicity and phylogenetic endemism. In this study, OCEUB uses as input six biodiversity dimensions, *i.e*. phylogenetic and species compositions, species richness and endemism, areas of endemism and phylogenetic endemism, for pinpointing relevant areas for biodiversity conservation alongside regions with marked knowledge shortfall across the Brazilian biomes.

## Results

**Figure 1:**
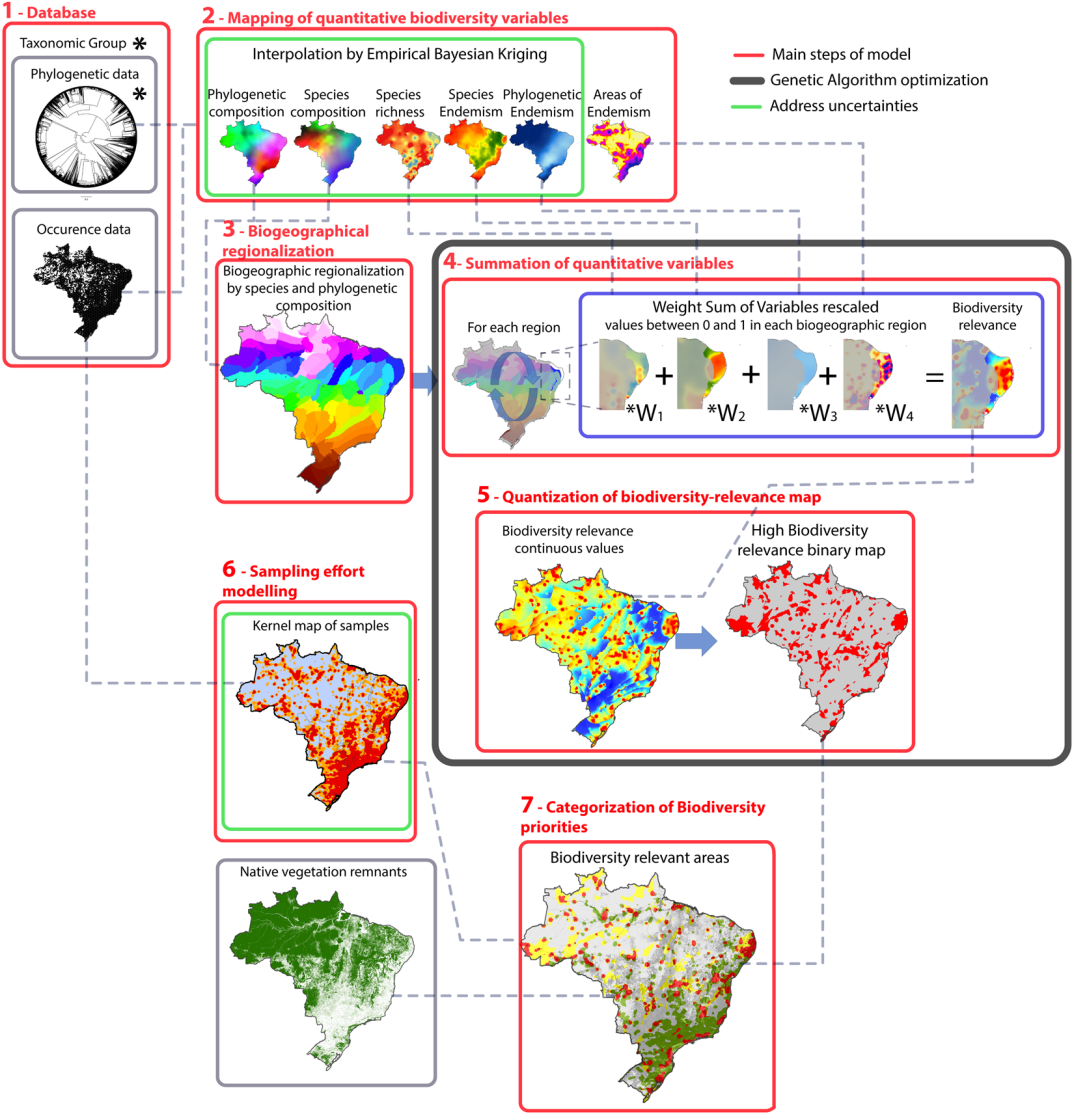
Modelling framework consists of seven main steps (red lines). (1) Database setting-up and verification, (2) mapping of quantitative biodiversity variables by using Empirical Bayesian Kriging, (3) biogeographical regionalization to define regions of unique biota, (4) summation of quantitative variables rescaled from 0 to 1 within each unique biota, (5) quantization of biodiversity-relevance map, (6) modelling sampling effort, and (7) categorization of biodiversity priorities (see Fig. 2). Modules circulated by dark lines include GA optimization and the ones by green lines address sampling uncertainty. Map created using Dinamica EGO (https://dinamicaego.com/).

OCEUB consists of main seven steps (Fig. 1). (1) Firstly, we set up a database of 2.3 million records of vertebrate, arthropod and angiosperm species for Brazil. From this collection, we selected 1,144,629 records that passed a rigorous check for geographic accuracy to assess the sampling effort and 882,468 records that also had taxonomic valid names to generate the biodiversity variables. We also built a phylogenetic supertree with 3,341 terminals (Material and Methods, Step 1, Appendix S2). (2) Because species occurrence records present gaps and sampling bias towards more accessible areas^5^, the model applies, as a second step, the empirical Bayesian kriging^16^ —a technique that reduces the effect of irregular sampling—to spatially interpolate the quantitative biodiversity variables. Modelled variables include the following biological dimensions: (*i*) species composition (β-diversity), (*ii*) phylogenetic composition (Phylogenetic β-phylodiversity), (*iii*) species richness, (*iv*) species endemism, *v*) phylogenetic endemism (Material and Methods step 2, Appendix S2). Specifically, for mapping areas of endemism, the model employs the Geographic Interpolation of Endemism (GIE)^17^. (3) Regions with distinct biota (unique combination of species/lineages) are not comparable because each biota is irreplaceable, *i.e*. do not occur elsewhere. Therefore, to map biodiversity relevant areas, our model stratifies Brazil’s territory into regions. Regionalization efficiently replaces complementarity analysis^18^, solving potential problems related to the use of this method in regions with sampling deficiency. We used species (β-diversity) and phylogenetic (β-phylodiversity) compositions to identify biogeographic regions with unique combination of species and lineages (Material and Methods, Step 3, Appendix S2). (4) Next, for each biogeographic unit from step (3), the model sums the quantitative biodiversity variables (species richness, species endemism, areas of endemism and phylogenetic endemism) after rescaling their minimum and maximum values within each region to 0 and 1. The model employs a weighted sum with the variable weights determined by using a Genetic Algorithm (GA) tool available in Dinamica EGO software^19^ (Material and Methods, Step 4, Appendix S2). (5) Conjointly with the optimization of step 4, the GA tool identifies a quantization threshold to classify the resulting summation of biodiversity variables into a binary map of areas of high and low biodiversity relevance (Material and Methods step 5, Appendix S2). (6) OCEUB also measures the sampling effort in order to estimate uncertainty, hence explicitly depicting areas of insufficient biodiversity knowledge. To this end, the model derives a kernel-interpolated density by using 1,144,629 records of species as a surrogate for the sampling effort (Material and Methods Step 6, Appendix S2). (7) Finally, the model adds the sampling effort and stamps the native vegetation remnants to generate the final set of classes that combine biological relevance, sampling density, and regional vegetation coverage. Hence, the biodiversity relevant map integrates the level of knowledge on the region’s biota with its degree of biological relevance and level of vegetation fragmentation (Material and Methods Step 7, Appendix S2).

To find the highly biodiverse areas, OCEUB seeks a compromise between the size of the area being prioritized and the largest biodiversity comprised within it. The optimization method applies a GA tool. GA is a heuristic method that mimics biological evolution by employing a natural selection algorithm to search for one of the best solutions for a specific objective or fitness function^20^. GA emulates natural evolutionary processes (selection, mutation, and recombination through crossover) to select over several generations of individuals, each one representing a combination of values for a set of selected variables (thus translated as a genetic sequence), an approximate optimal solution for a complex problem. In our case, the fitness function is a ratio between the number of species plus species endemism, phylogenetic diversity and phylogenetic endemism encompassed by the area being prioritized and its geographic extent (Material and Methods, GA).

**Figure 2:**
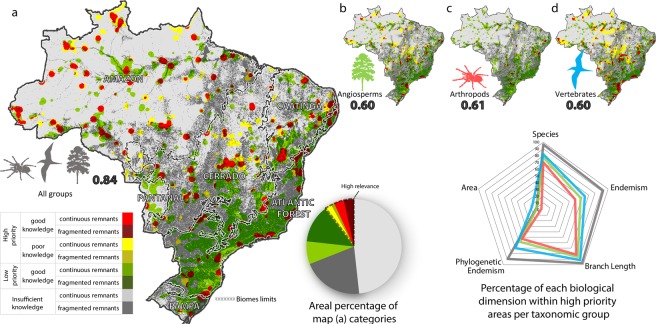
Relevant areas for biodiversity conservation: (**a**) based on all taxonomic groups, (**b**) only on angiosperms, (**c**) only on arthropods, (**d**) only on vertebrates. Bold numbers indicate model performance. Colours in pie chart represent map (**a**) categories. Colours in radar chart represent taxonomic group, the vertices represent the dimensions of biodiversity analysed in the study. The closer the vertex is to the line, the more this dimension is being covered (in percentage). Map created using Dinamica EGO (https://dinamicaego.com/).

As a result, OCEUB attained, as an optimal solution, a model able to encompass a large portion of Brazil’s known biodiversity (96% of species, 97% of lineages, 89% of phylogenetic endemism and 92% of endemism) in only 10% of the country (Fig. 2). This model scores a fitness of 0.84 out of 1 (Material and Methods GA), by weighting most heavily the areas of endemism (0.94), followed by species richness and endemicity (0.57 and 0.54 respectively) and phylogenetic endemism with 0.17 (Appendix S1). Of the highly relevant areas, 43% lie in the Amazon, 25% in the Cerrado, 17% in the Atlantic Forest, 11% in the Caatinga, 2% in the Pampa and 1% in the Pantanal. Whereas 80% of the Amazon relevant areas constitute large and continuous expanses of forests, 26% of which are protected by conservation units and indigenous lands, roughly 80% of the relevant areas in the Atlantic Forest consist of highly fragmented forest remnants, of which only 3% are located in protected areas. The other biomes, in turn, characterize intermediate situations.

We estimated model uncertainty by measuring the sampling effort. In addition, collection gaps and difference in sampling effort were minimized by spatially interpolating the quantitative biodiversity variables and resampling of species richness (Appendix S2). The effectiveness of the applied techniques in minimizing the effects of sampling effort is evidenced by the low correlation between the maps of the modelled biodiversity variables and the sampling effort (0.04 for species richness, −0.24 for phylogenetic endemism, 0.09 for endemicity and 0.4 for areas of endemism). Although our method was able to identify areas of lower biodiversity priority within regions of good sampling as well as high priority within poorly sampled regions (Fig. 2), roughly 69% of Brazil’s territory presents insufficient information for identifying biodiversity priority areas. Our current biodiversity knowledge is therefore insufficient in 82% of the Amazon biome and as well as in large portions of the other biomes (64% in Caatinga, 68%, in Cerrado, 60% in Pampa, and 60% in Pantanal). In turn, the Atlantic Forest is the only biome that contains a reasonable sampling covering 73% of its expanses.

To analyse the effectiveness of using taxonomic groups as surrogates for identifying biodiversity priority areas (Surrogate Test in Material and Methods), we built models based only on a single group (angiosperms, arthropods and vertebrates). Nonetheless, the fitness function for optimizing these models employs data from all groups. This procedure ensures that the model based solely on one group be the best predictor for the geographic distribution of all groups, yielding thus the best surrogate model.

Our best models based only on a single group were less efficient than the model with all taxonomic groups (Fig. 2 and Appendix S1). The model based only on vertebrate data encompasses more species, phylogenetic diversity, endemism and phylogenetic endemism than the other surrogates models (Fig. 2). However, highly biodiverse areas in this model encompass a larger portion of the Brazilian territory (7% more) than those of other surrogate models as well as that of the model with all groups (Fig. 2 and Appendix S1). The vertebrate-based model comprises a considerably smaller share of biodiversity than that of the model with all groups (−14% species, −28% endemism, −5% phylogenetic diversity, and −20% phylogenetic endemism (Fig. 2 and Appendix S1). The model based on angiosperm data encompasses −19% of the species, −35% of endemism, −11% of phylogenetic diversity and −31% of phylogenetic endemism than the model based on all data. In turn, the arthropod model contains −26% species, −43% endemism, −15% phylogenetic diversity, and −36% phylogenetic endemism than the model based on all groups. We also tested the relevance of including phylogenetic data into our prioritization modelling. To do so, we ran a model without phylogenetic data and evaluated its ability to pinpoint highly biodiverse areas (even including phylogenetics). As a result, this model scored 0.67, performing 20% worse than the best model (Fig. 2).

## Discussion

OCEUB successfully pinpoints small highly biodiverse areas alongside regions with knowledge shortfalls, hence explicitly addressing regions where evidence-based conservation decisions are not possible yet. These regions should thus be aimed for sampling campaigns. Additionally, the GA best solution emphasises the need to include the variable “area of endemism” in biodiversity prioritization studies. Although theoretical studies have highlighted the relevance of this variable^21^, none had before demonstrated this importance empirically.

The GA optimization indicates that the use of only one or a few taxonomic groups, such as mammals and birds, is not sufficient for identifying relevant areas for biodiversity conservation in a megadiverse country like Brazil (Appendix S3). Our models based only on a single group, such as vertebrates, were less efficient than the one that uses information from all taxonomic groups (Appendix S1). Moreover, sampling gaps, hence knowledge shortfalls, are similar for all groups^5^, making the use of taxonomic surrogates (such as birds or mammals) ill-suited to infer the geographic distribution of other groups. These results underline the multidimensional aspect of biodiversity.

Conversely to many previous studies^10,11^, we did not observe significant differences in using surrogates from different taxonomic groups (Fig. 2, Appendix S1). The model based only on vertebrate’s data encompasses a larger percentage of the biodiversity dimensions (Fig. 2), but this is due to its larger area prioritized (17%) compared to those of other surrogate models. In turn, the model based only on vertebrates is more efficient in protecting lineages in relation to other surrogate models, probably because of its largest share of phylogenetic data, since 74% of the terminals of the supertree are vertebrates (Fig. S1). This relative abundance in fact influences our fitness function; thus to allow comparison, we always included in the fitness function data from all groups in order to evaluate any model run (Appendix S1).

Biodiversity conservation focuses on the protection of species. However, approaches that do not consider phylogenetic data are not effective in protecting evolutionary lineages, which are essential to preserve the history of life as well as the broad range of ecosystem services provided by biodiversity^22,23^. Note that excluding phylogenetic data impaired model performance by 20% in relation to the best model, indicating the importance of this dimension to biodiversity modelling (Appendix S3). Although we have assembled the largest phylogenetic dataset for Brazil to date, the developed phylogenetic supertree contains only 14% of the overall set of species. Most of the data are for vertebrates; arthropods are very poorly represented and angiosperms are in an intermediate situation. The reduction of this Darwinian shortfall^24^, should be therefore a top priority for biodiversity surveys in tropics.

The general lack of biological knowledge^5,25,26^ together with the high deforestation threat still posed to the Brazilian biomes^27–30^ call for the need to develop differentiated conservation strategies across the country. A comprehensive conservation planning should target the expansion of conservation units to protect the last continuous expanses of native vegetation rich in biodiversity, such as the ones of the Amazon forest, while policies to solve Brazils’ forest code implementation^30,31^ are directed to restore the most biodiverse, but extremely fragmented landscapes of the Atlantic Forest. By highlighting the major biological relevant areas along with regions where further inventories are needed, our results, along with the knowledge about conservation gaps^26^, represent a first step towards a systematic conservation planning for Brazil. Such planning should also include the opportunities and costs for conservation, such as land designation^32^, current and future agricultural lands, hence land-use prices, deforestation threat^31^, land-use policies^30^ as well as climate change. All these variables could also be included in our modelling approach to seek a comprehensive and balanced conservation strategy.

## Material and Methods

OCEUB consists of main seven steps (Fig. 1): (1) Database setting-up and verification, (2) mapping of quantitative biodiversity variables, (3) biogeographical regionalization, (4) summation of quantitative variables, (5) quantization of biodiversity-relevance map, (6) sampling effort modelling, and (7) categorization of biodiversity priorities. In addition to these modelling components, we test the use of surrogates and exclusion of phylogenetic data. To optimize OCEUB, we apply a GA tool to determine the weights for summing the quantitative variables, after rescaling their values in each biogeographical region from 0 to 1, and to define the quantization threshold to classify the map resulting from the summation process into two classes of biodiversity relevance (high and low).

### Fitness function and Genetic Algorithm optimization

Our fitness or objective function aims to find the smallest possible areas that encompass most biodiversity. Specifically, it comprises the number of species (*r*) plus the number of lineages—lengths of branches (*pl*)—, endemicity—sum of WE index of species (*e*)—, and phylogenetic endemism—the sum of PE index of species (*pe)―* divided by four minus the ratio between the extent of areas of high biodiversity relevance (*a*) and the total area under analysis—i.e. Brazil (*A*) (Equation 1). Note that all variables are rescaled between 0 and 1 in each biogeographic region. As a result, a model that encompasses the maximum number of species, lineages, endemicity, and phylogenetic endemism in the smallest possible area would yield a value close to 1.

1 $$(\frac{{\rm{r}}+{\rm{pl}}+{\rm{e}}+{\rm{pe}}}{4})-{\rm{a}}/{\rm{A}}$$

The GA optimization simultaneously looks for optimal values for the weights in the summation of quantitative biodiversity variables (step 4) and the choice of the quantization threshold (step 5). In the GA process, each individual (particular model) is composed of genes (in our case, a sequence of five alleles representing the values of the four weights and the quantization threshold); our primeval gene is set to 0.5, 0.5, 0.5, 0.5, 0.5. GA begins by randomly generating an initial population of 20 individuals using as mould the primeval gene, *i.e*. max and min bounds set to 1 and 0, respectively. Each individual or model is run and then ranked according to its fitness (equation 1). The algorithm selects the top-ranked ones plus a smaller set of individuals randomly picked. This latter selection is necessary to obtain a diversified population. Next GA produces a new generation by mutating and recombining (crossover) the genes of the selected individual. The GA algorithm is set to iterate up to 100 generations or to stop if an asymptotic solution is attained.

### Step 1: Database setting-up and verification

From a collection of 2.3 million records of vertebrate, arthropod, and angiosperm species for Brazil, we selected 1,144,629 that passed a rigorous check of geographic accuracy to assess the sampling effort and 882,468 records that also had taxonomically valid names to generate the biodiversity variables (Appendix S3). We also built a phylogenetic supertree with 3,341 terminals (Fig. S1 in Appendix S2) by pruning the tree of Hinchliff *et al*.^33^.

Our species distribution database includes only the most specious and widely distributed angiosperm families in Brazil: *Asteraceae, Bromeliaceae*, *Fabaceae*, *Melastomataceae*, *Myrtaceae, Orchidaceae, Poaceae* and *Rubiaceae*. For arthropods, we compiled data on bees, spiders, polydesmid millipedes, flies, tiger moths, dragonflies, and *Orthoptera*. Vertebrates include birds, mammals, and amphibians. Occurrence data come from the following online databases: GBIF (http://www.gbif.org); CRIA (http://www.splink.org.br); Herpnet (http://www.herpnet.org); Nature Serve (http://www.natureserve.org); and Orthoptera Species File (orthoptera.speciesfile.org) (Appendix S1). Access date is December 2014. The names of the taxonomic groups and the location filter “Brazil” were used in all the search queries. We also compiled data from taxonomic literature and biodiversity inventories. All data were checked for geographic accuracy by crossing the municipality registered in the occurrence record with a map of Brazilian municipalities (http://mapas.ibge.gov.br). Records that lacked geographic coordinates or presented location errors were georeferenced using the Brazilian municipality map and locality information. All data were verified for the validity of taxonomic names through specific catalogues (Appendix S3) and were directly reviewed by experts of each group (Appendix S1). Comparative analyses were performed for the whole dataset and separately for each data partition (angiosperms, arthropods, and vertebrates).

In order to identify geographic patterns of evolutionary lineages, we compiled phylogenetic trees of taxa with geographic distribution limited to Brazil (Fig. S1, Appendix S2). The phylogenetic trees were converted from published figures to newick format using TreeSnatcherPlus software^34^. In addition, we used the phylogenetic data from The Open Tree of Life platform^33^ assembled from empirical phylogenetic studies (thus excluding branches located based on taxonomic classification). Since branch lengths are not comparable between different trees and are sometimes not available, we considered all branches with length equal to one. The trees were merged into a supertree by matrix representation with parsimony^35^.

### Step 2: Mapping of quantitative biodiversity variables

Using our collection of species records, we derived the following variables: endemicity, phylogenetic endemism, areas of endemism, and species richness. To check redundancy between the quantitative variables, we performed pairwise correlation tests using the Pearson correlation with corrected degrees of freedom^36^ (Appendix S2). Thus, we discarded the use of phylogenetic diversity as an input variable because of its high correlation with species richness (see Appendix S2). Functions that calculate these variables are available in the BioDinamica package of Dinamica-EGO freeware (http://dinamicaego.com/).

#### Endemicity

For mapping endemicity (the predominance of species with restricted geographic distribution), we used the index of Weighted Endemism (WE)^37^. This index yields a value for each species that is equal to the inverse of the species’ distribution area. The endemism is expressed as the sum of WE index for each 1° hexagonal cell (Fig. S6, Appendix S2).

#### Phylogenetic endemism

To identify geographic patterns of phylogenetic endemism (predominance of evolutionary lineages restricted to a specific region), we used the index of Phylogenetic Weighted Endemism (PWE)^38^. Phylogenetic endemism is interpolated into 1° hexagons (Fig. S8 in Appendix S2).

#### Species richness

For quantifying alpha diversity (species richness), we implemented a spatial resampling method. Species richness (the number of species per unit area) is the biodiversity variable most influenced by sampling effort^5^. Hence, the most direct way of estimating species richness, *i.e*. summing the number of species per sampling unit (squares or hexagons), may produce unrealistic spatial patterns. Alternatively some studies apply species distribution modelling (SDM) to estimate the distribution of species, and sum the resulting distribution maps to obtain a species richness model^39,40^. However, this approach may also be biased due to the influence of the collection effort (Appendix S2). To overcome this problem, we applied a set of resampling techniques to generate a uniform sampling distribution throughout the study area, thus reducing the effect of the collection effort on the species richness. To simulate a uniform distribution of sampling, we used a fixed number of records within each hexagon. We performed tests to select the numbers of samples and sub-samples in order to minimize the effect of the sampling bias (Appendix S2). From these tests, we chose 50 records per sample (hexagon) and randomly selected a subsample (25%) of records in the hexagon samples (Fig. S11, Appendix S2). In this way, all hexagons in the study area have the same number of subsampled records. In each hexagon, we counted the number of species in the subsample. This procedure is repeated 1,000 times and the average of the species richness is recorded for each hexagon (Fig. S11, Appendix S2). As a result, we obtained the relative values of species richness based on a uniform distribution of sampling throughout the study area, thus largely reducing the effect of the sampling effort on mapping species richness. Finally, we spatially interpolated the results from the latter step by using Empirical Bayesian Kriging technique (see below interpolation method).

#### Area of endemism

To identify areas of endemism patterns, we used Geographic Interpolation of Endemism (GIE)^17^. Areas of endemism consists of geographic regions that present species with high distributional congruence (highly sympatric species)^21^. These areas have a unique evolutionary and ecological history and thus are of high relevance for biodiversity conservation^21^. To apply the GIE method, we first classified species into nine groups according to the distance between the centroid of each species geographic distribution and their farthest occurrence point delineated as such: up to 50 km; 51–200; 201–400; 401–600; 601–800; 801–1,000; 1,001–1,500; 1,501–2,000, and 2,001–3,299 km. To generate the consensus of AoEs (Fig. S7 in Appendix S2) we rescaled the resulting map into 0 and 1. For more details on the GIE method, see Oliveira *et al*.^17^.

#### Interpolation method

In order to minimize the effects of sampling gaps and collection bias, we spatially interpolated all biological variables but areas of endemism by using Empirical Bayesian Kriging technique. This technique considers that intermediate values occur proportionally to the distance between observed points following a smoothed distribution curve^16^. Additionally, this interpolation method solves the problem of choosing the model parameters, since it automatically calculates the best parameters by using sub-setting and bootstrap simulation. In this way, this technique disregards values that are not expected given a spatial autocorrelation function. Hence, samples that present disparate values due to small number of occurrences can be automatically discarded. We have chosen this interpolation because of these advantages and its straightforward premise (spatial autocorrelation), which is adequate for the interpolation of biodiversity variables. In order to verify the effectiveness of our method in minimizing sampling effort differences in the maps of quantitative variables, we performed a correlation analysis (Pearson with degrees of freedom corrected by spatial autocorrelation) between the sampling effort map and those of the biodiversity variables: species richness, weight endemism index, phylogenetic endemism and areas of endemism.

### Step 3: Biogeographical regionalization

To ensure complementarity of the areas selected by the model, we performed a biogeographic regionalization of the study area. The procedure guarantees that each region contains a unique biota in terms of species and evolutionary lineages, *i.e*. each region has much greater homogeneity in relation to species composition and phylogenetic composition when compared with its exterior.

To produce the map of biogeographic regions, we carried out an unsupervised classification of species composition and phylogenetic composition maps (Fig. S4, Appendix S2). We used the maximum likelihood algorithm to classify maps of species and phylogenetic composition (see Appendix S2 for details about determination of number of classes). For building the maps of species and phylogenetic composition, we used the Interpolation of Species Composition - SCI^41^ and the Interpolation of Phylogenetic Composition - IPC (an adaptation of the previous method for mapping phylogenetic beta-diversity patterns by PhyloSor index^42^). In these processes, we used a hexagonal grid of 1° as the sample unit. We ran 10,000 NDMS (non-metric multidimensional scaling) rounds to get the best fit. Detailed description of this method is provided by Oliveira *et al*.^41^. This method is also available in BioDinamica package of the Dinamica-EGO freeware ((http://dinamicaego.com/).

### Step 4: Summation of quantitative variables

We performed a weighted sum of the selected quantitative variables (step 2) after rescaling their minimum and maximum values within each biographic region to 0 and 1 (step 3). In this process, we applied the GA tool to determine the values for the weights in the summation that maximize our fitness function (Fig. 1). The result of the weighted sum is a map of continuous values of biological relevance varying from 0 to 1.

### Step 5: Quantization of biodiversity-relevance map

We applied a threshold to discretize the map into two categories: relevant and non-relevant areas. The determination of the optimal threshold was performed simultaneously with step 4 through the GA tool.

### Step 6: Sampling effort modelling

We modelled the sampling effort to incorporate uncertainties inherent to collection gaps and sampling bias. To do so, we derived the kernel-interpolated density of all records of species occurrence: 1,144,629 records (Fig. S9, Appendix S2). In the Kernel interpolation, we used the value calculated from the spatial variant of Silverman’s Rule of Thumb^43^, implemented in ArcGIS, as the area of influence around each species record. This procedure provides an approximation of the Gaussian distribution for the distances from interpolated points.

### Step 7: Categorization of Biodiversity priorities

To obtain the final map of biodiversity priorities, we merged the sampling effort and stamped the native vegetation remnants on the resulting binary map of biodiversity relevance (step 5). Hence, the biodiversity relevant map combines the level of knowledge on the region’s biota with its degree of biological relevance, and level of vegetation fragmentation.

### Testing surrogate models and exclusion of phylogenetic data

#### Taxonomic Surrogate

To analyse the effectiveness of using taxonomic groups as surrogates for identifying biodiversity priority areas, we built models based only on a single group (angiosperms, arthropods and vertebrates). The surrogate models were built using the same procedures described above; nevertheless, the fitness function for optimizing these models employs data from all groups. This procedure ensures that the model based solely on a single group be the best predictor for the geographic distribution of all groups, yielding thus the best surrogate model. Finally, we compared model performance by using our fitness function.

#### Relevance of Phylogenetic data

To evaluate the use of phylogenetic data into biodiversity prioritization modelling, we ran a model without phylogenetic data and evaluated its ability to pinpoint highly biodiverse areas (even including phylogenetics). Thus, this model did not employ the phylogenetic composition in the biogeographic regionalization (step 3), nor did it use the phylogenetic endemism as a quantitative variable. However, we tested the ability of this model to encompass all biodiversity dimensions including the number of species, lineages, endemism and the phylogenetic endemism. All models and input data are available at http://csr.ufmg.br/bioconservation/.

## Supplementary information

Appendix S1

Appendix S2

Appendix S3
