# Supplementary material for: Modelling Highly Biodiverse Areas in Brazil
Source: Sci Rep. 2019 Apr 23;9:6355. doi: 10.1038/s41598-019-42881-9 (PMC6479156; doi:10.1038/s41598-019-42881-9)
Supplement: Supplementary file 2 — Appendix S2 [file 41598_2019_42881_MOESM2_ESM.docx]

**Support Information S2**

**Modelling Highly Biodiverse Areas in Brazil**

**Detailed Material and Methods**

Ubirajara Oliveira, Britaldo Silveira Soares-Filho, Adalberto J. Santos, Adriano Pereira Paglia, Antonio D. Brescovit, Claudio J. B. de Carvalho, Daniel Paiva Silva, Daniella T. Rezende, Felipe Sá Fortes Leite, João Aguiar Nogueira Batista, João Paulo Peixoto Pena Barbosa, João Renato Stehmann, John S. Ascher, Marcelo F. Vasconcelos, Paulo De Marco, Peter Löwenberg-Neto, Viviane Gianluppi Ferro.

1. **Details of Material and Methods and supplementary figures**


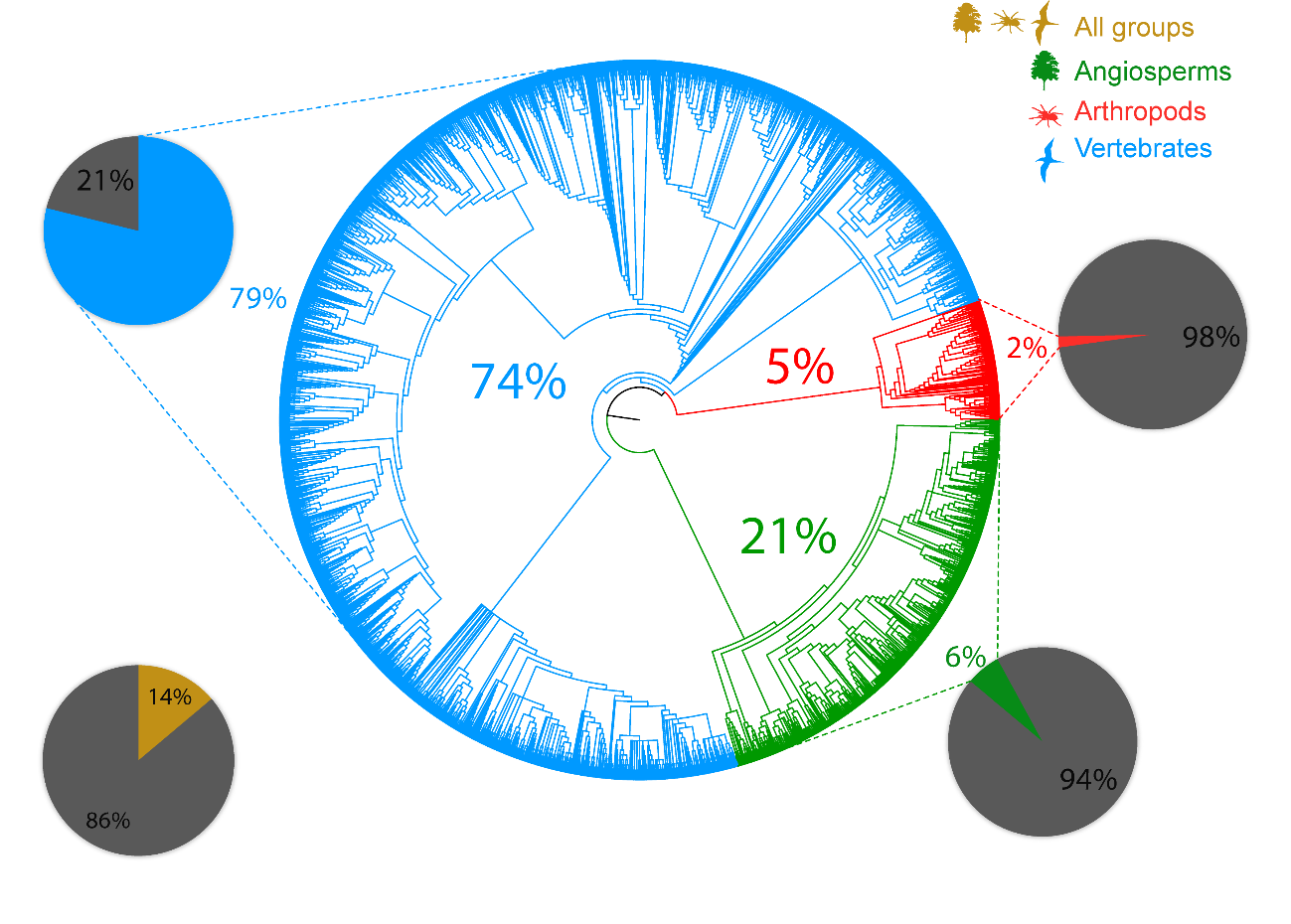


**Figure S1**: Phylogenetic supertree with 3,355 species terminals (Appendix S4). The pie charts show the proportion of species from the species distribution database represented in the supertree. The numbers in the tree represent the percentage of each group in the supertree.


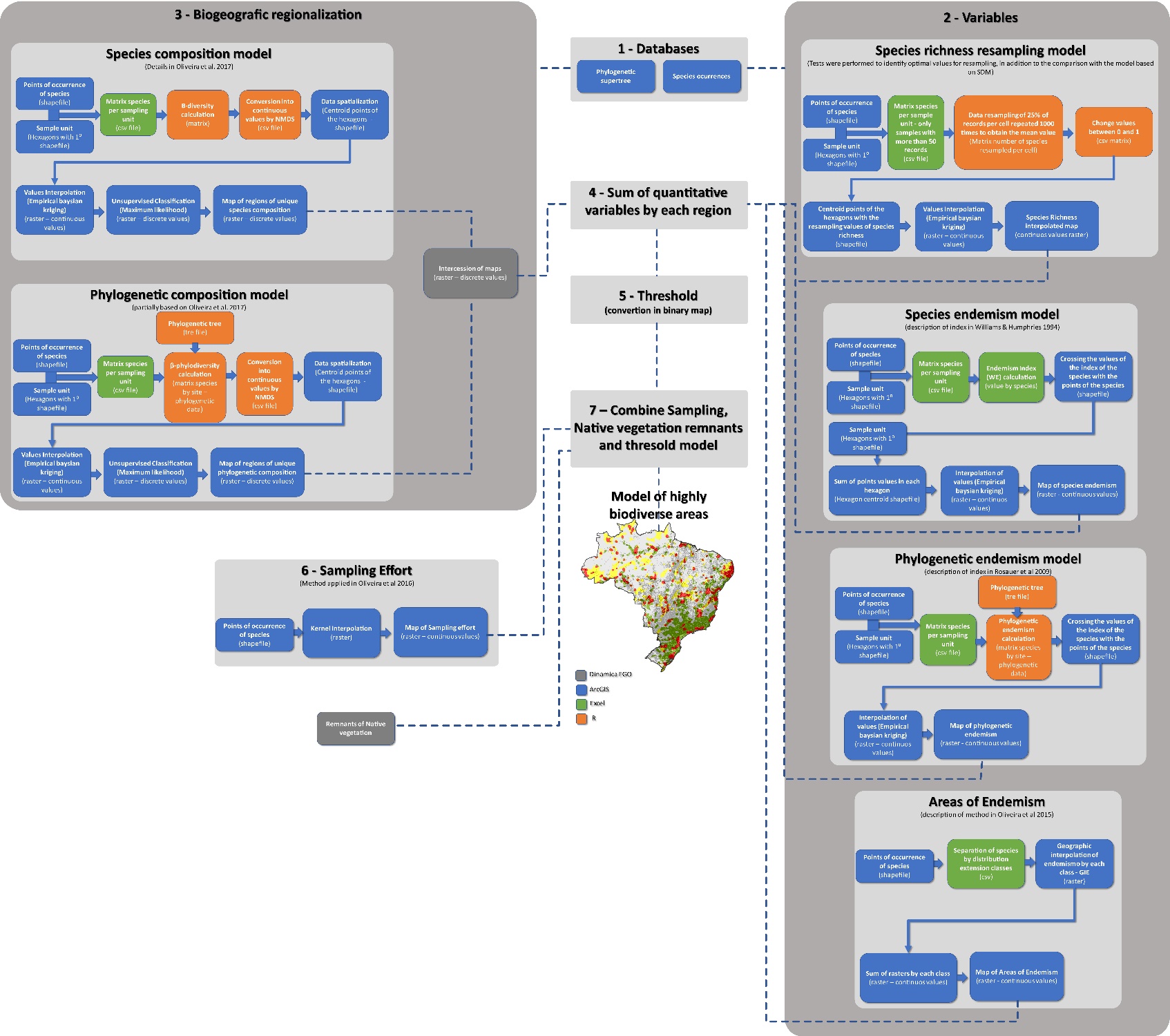


**Figure S2**: Detailed model of relevant areas for biodiversity conservation.

***Classes in Biogeographic regionalization***

To determine the ideal number of areas (classes) for performing unsupervised classification, we ran the classification algorithm several times varying the number of classes, the number of classes from 2 to 60, until the final obtained number of classes had stabilized (see the final values of number of classes in the Figure S3). As a last step, we combine the classified maps of species and phylogenetic composition to obtain the regionalization map.


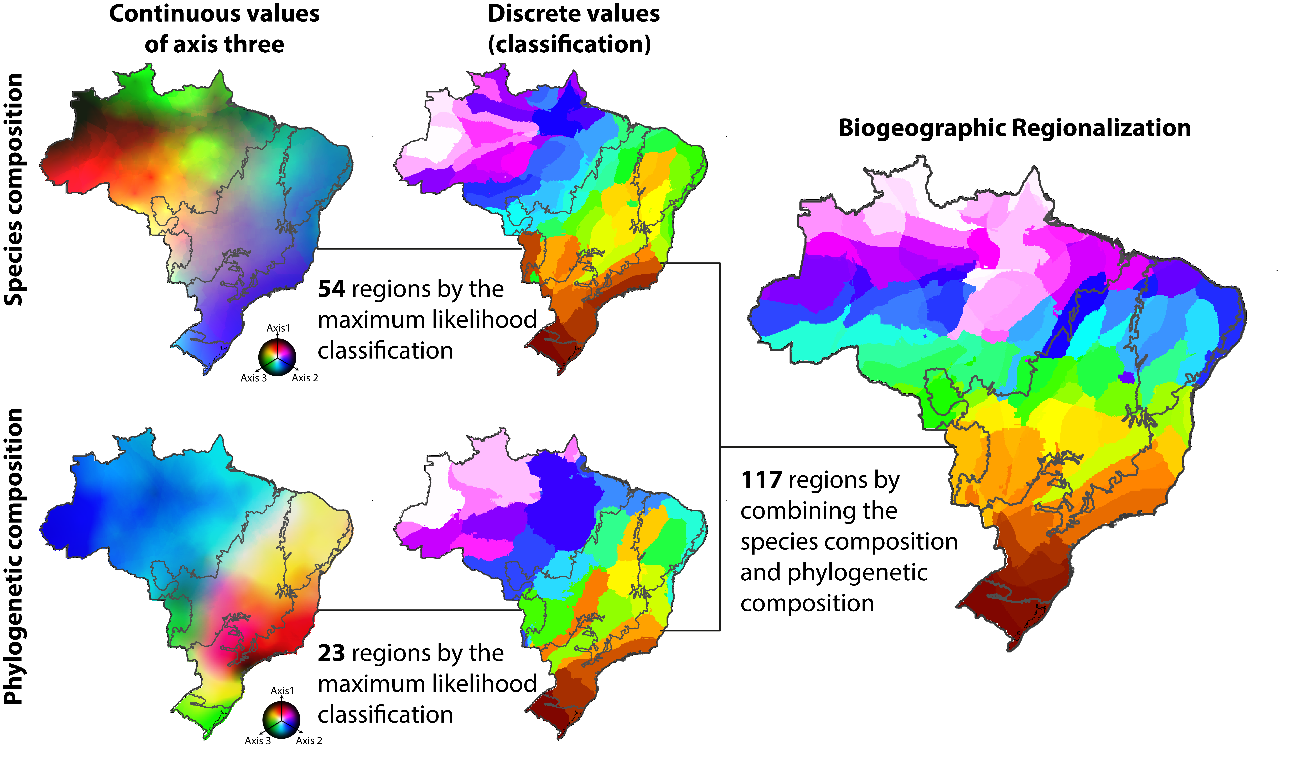


**Figure S3**: Regionalization of species and phylogenetic composition. The unities are produced by unsupervised classification through a maximum likelihood algorithm based on tree axes of the NMDS species composition map generated as in the Figure S2. The regionalization map was obtained by combine the maps of classified species and phylogenetic compositions. Dark lines indicate biomes boundaries. *Map created in Dinamica EGO (https://csr.ufmg.br/dinamica/).*

***Species Richness resampling parameters tests***

We performed sensitivity analyses for each parameter of the species richness model to investigate how the different parameters of the resampling we conducted might affect the results (Figure S4 and S5). To identify the best combination of parameters, we tested the correlation between the species richness output from models generated with each set of parameters and the sampling effort. The model that showed a lower ratio between sampling effort and the variable species richness was selected for the Bayesian Kriging interpolation.

*Test of sampling unit size:* We defined hexagonal partitions as sampling units. Since the size of the hexagons can influence the analysis, we tested the effect of using 1, 1.5, and 2 degrees hexagons.

*Test of sample size:* The number of records in the sample may also influence the results. Thus, we conducted tests with 50, 100 and 200 records per hexagon.

*Test of subsample size:* Again, the size of the subsamples can influence the results. In order to test this issue, we experimented 25, 50 or 75% of the hexagon sample.


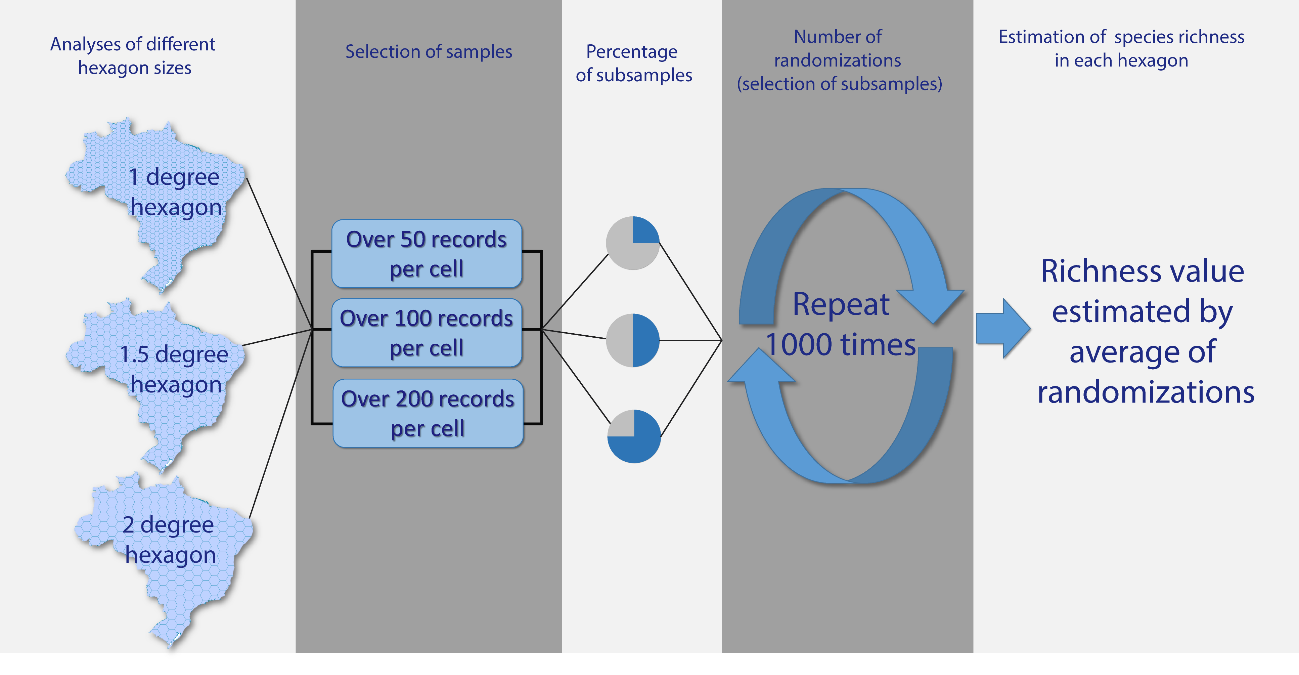


**Figure S4**. Steps of data resampling for mapping species richness and sensitivity tests for each parameter of species richness model.


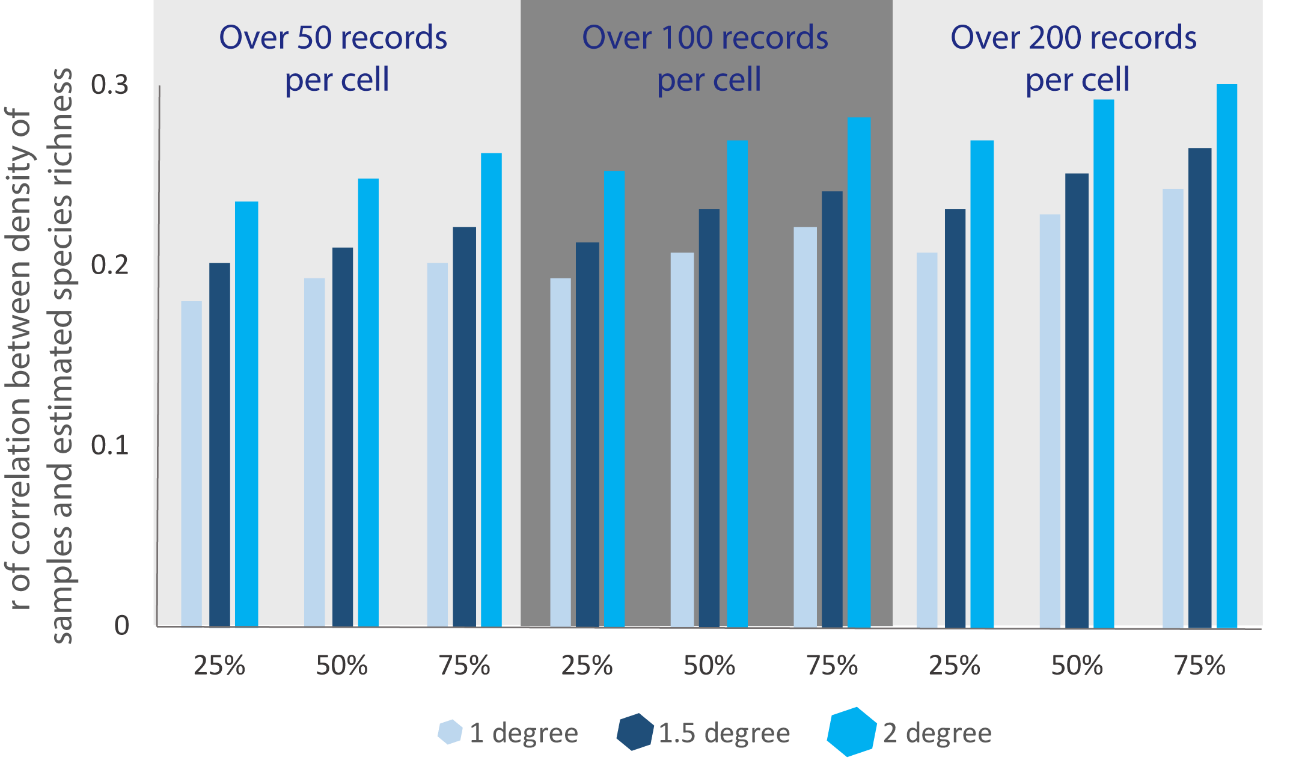


**Figure S5**. Correlation between different sampling design (sensitivity tests) and sampling effort (density of samples). The resampling procedure used for producing the species richness index has proven efficient in removing the sampling effect from this variable (r = -0.03). In addition, the different simulated sampling designs showed no significant differences in their results. Thus, we opted for interpolating the values obtained in the sampling of 50 records and 25% of sub-samples due to the lower effect of sampling effort.


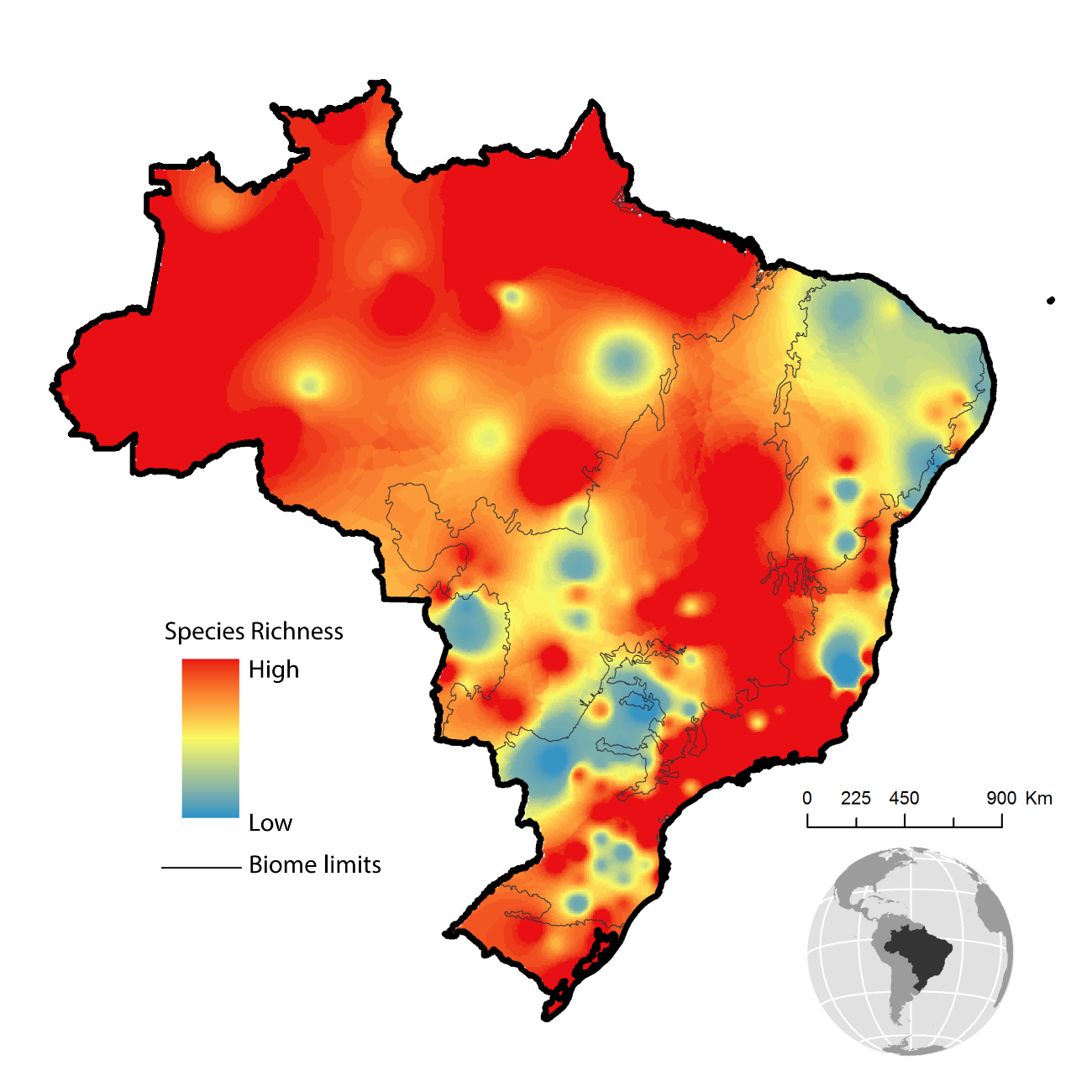


**Figure S6**. Species richness, rescaled between 0 and 1, based on resampling of records (sampling of 50 records and 25% of sub-samples). *Map created in Dinamica EGO (https://csr.ufmg.br/dinamica/).*

***Species richness model comparation: SDM and Resampling***

To compare our resampling technique for mapping species richness with those of SDMs we tested the relationship between the sampling bias by using a non-stationary model. The non-stationary model allows us to test changes in the dependence between variables across geographic regions. For our non-stationary model we used the Geographically Weighted Regression method (GWR). To quantify the effect of sampling bias and the Hutchinsonian shortfall on the SDM-based model of species richness we used as the response variable the density of records (sampling effort) and the Hutchinsonian shortfall map ^6^, respectively (Figure S7) . To check the effect of sampling bias on our Bayesian interpolation species richness model we used the density of records (sampling effort) as the response variable (Figure S10).

We built a species richness model based on SDM of 4,344 species. These species were used because of their larger number of records with high geographic accuracy. We only used species that presented more than 15 accurately georeferenced records in a specific locality (Appendix S3). To build the species richness map we integrated the results from all the variant models of species based on the following SDM algorithms: Bioclim, Domain, Mahalanobis distance, Maxent, Generalized Linear Model (GLM), Generalized Boosting Model (GBM or Boosted Regression Trees), and Support Vector Machine (SVM). The resulting model of species richness based on SDMs consists of an average of maps of species richness (from summing models of species from each algorithm). The SDMs were built based on the ﬁrst four axes of a Principal Component Analysis (PCA) (based on a correlation matrix) summarizing 19 bioclimatic (http://www.worldclim.org/) and two topographic variables: elevation (from Worldclim) and derived slope. We used the lowest value of suitability in training threshold points. The PCA was implemented in ArcGIS using 25-km^2^ spatial resolution. To exclude random models, we used the area under the curve (AUC) of pseudo absences and only included SDMs that present AUC above 0.7.


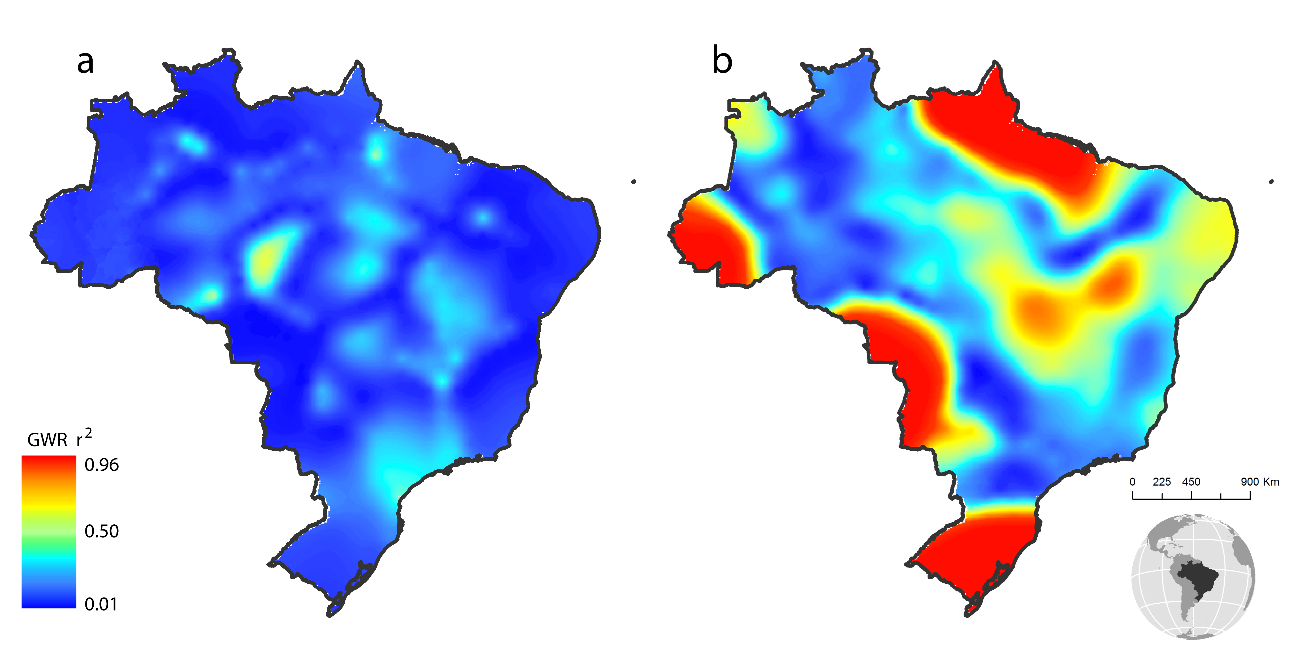


**Figure S7**. Results of GWR (r^2^) for species richness model based on (a) resampling and (b) Species Distribution Models (SDM). Warm colours show where correlation between the model and the collection sampling bias is stronger. *Map created in Dinamica EGO (https://csr.ufmg.br/dinamica/).*

***Correlation between quantitative variables***

To check redundancy between the quantitative variables, we performed correlation tests between the variables through the Pearson correlation with corrected degrees of freedom^13^ (Table S1). To verify the relationship between species richness and phylogenetic diversity, we performed a linear regression (Figure S8).

Table S1 - Correlation between variables.

|  | **Areas of Endemism** | **Endemism Index** | **Phylogenetic**  **Endemism** | **Species Richness** |
| --- | --- | --- | --- | --- |
| Areas of Endemism |  | 0.13 | 0.001 | 0.12 |
| Endemism Index |  |  | 0.63 | 0.40 |
| Phylogenetic Endemism |  |  |  | 0.43 |

**Figure S8**. Results of regression between species richness and phylogenetic diversity.


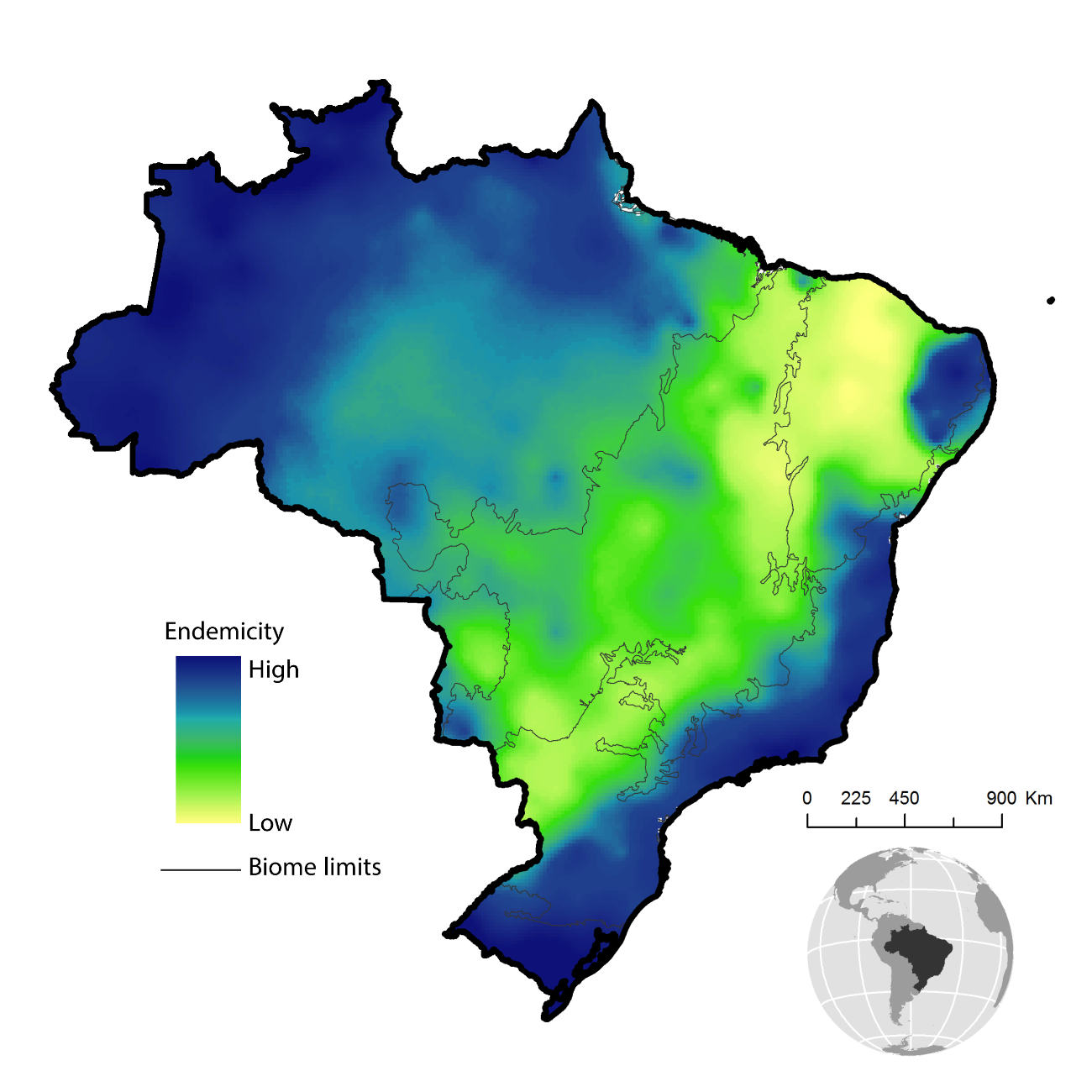


**Figure S9** - Interpolated index of Weighted Endemism. The blue shades indicate areas with the larger number of species with restricted geographic range. *Map created in Dinamica EGO (https://csr.ufmg.br/dinamica/).*


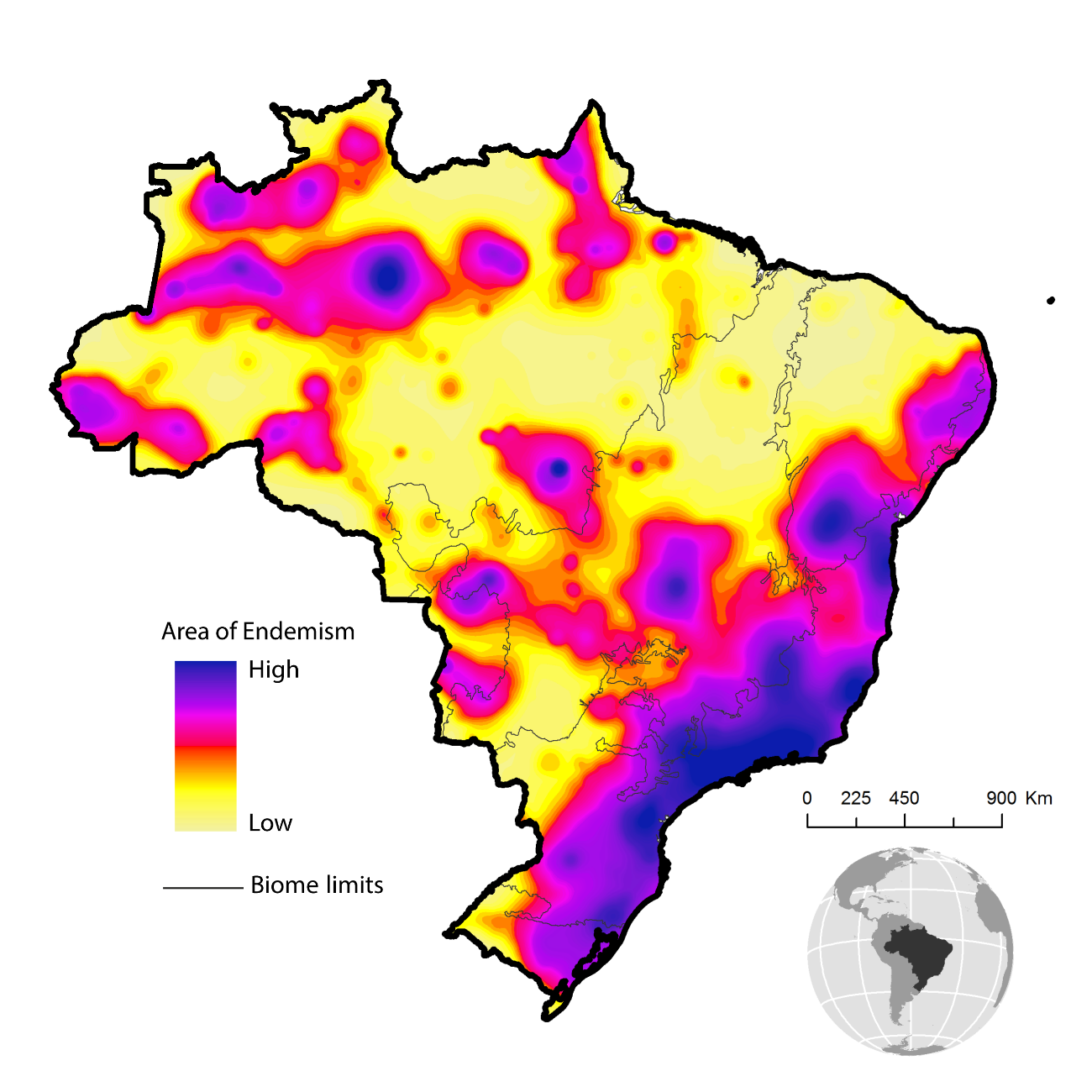


**Figure S10** - Consensus of areas of endemism. The purple shades indicate areas of endemism with the maximum number of species as well as with the higher distributional congruence. The areas shaded in yellow neither have neither data nor are areas of endemism. *Map created in Dinamica EGO (https://csr.ufmg.br/dinamica/).*


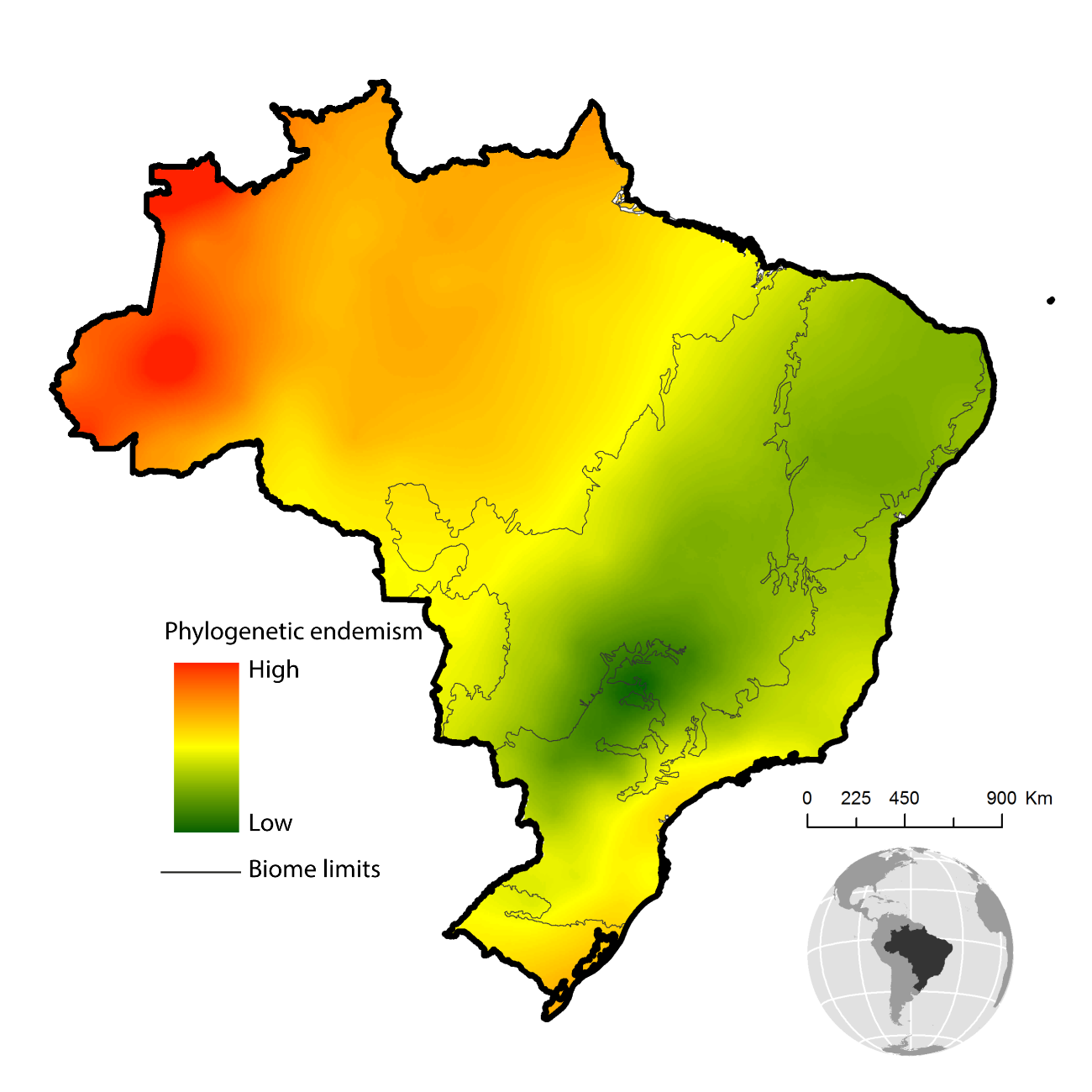


**Figure S11**. Index of Phylogenetic Weighted Endemism. The red shades indicate areas with the larger number of phylogenetic lineages with restricted geographic range. *Map created in Dinamica EGO (https://csr.ufmg.br/dinamica/).*


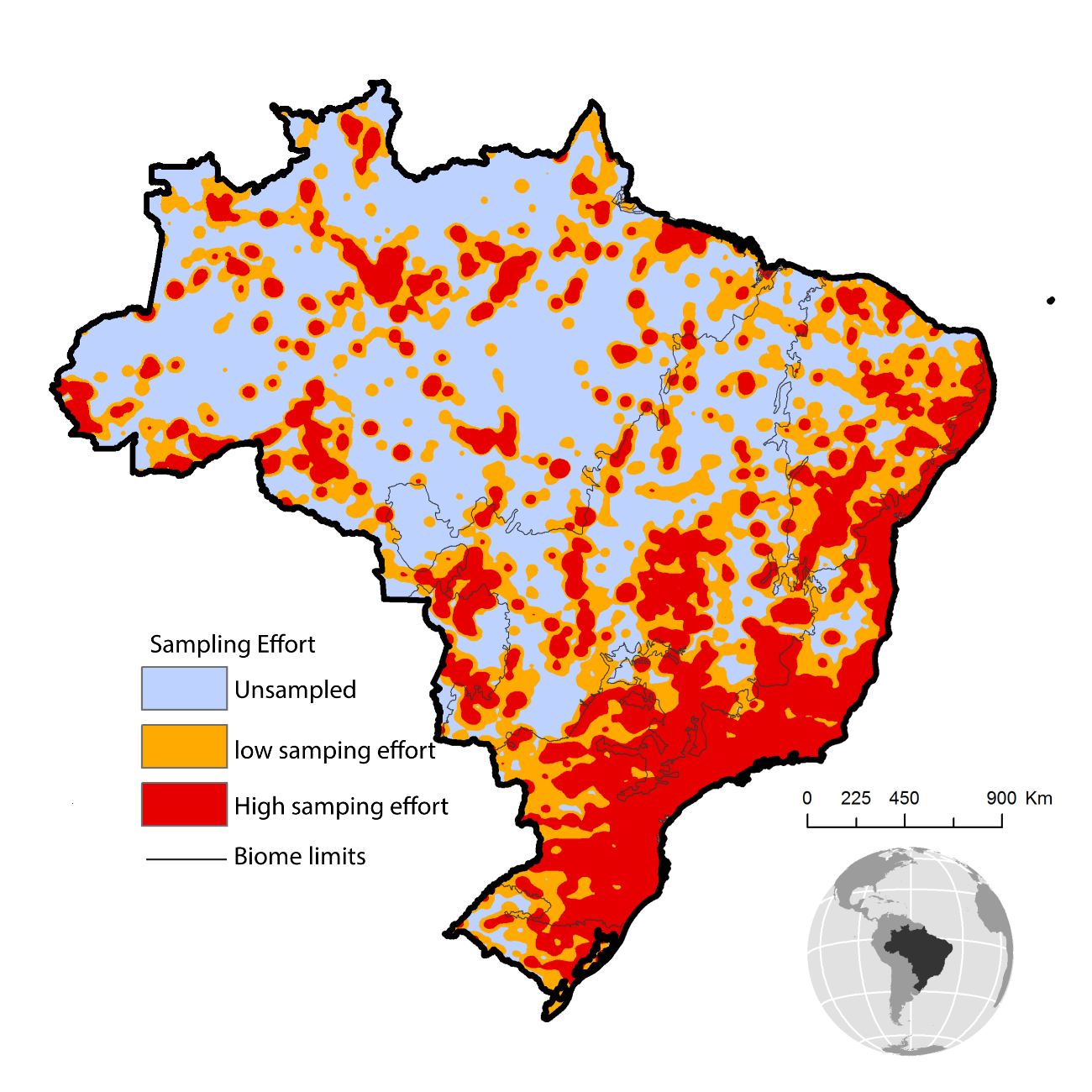


**Figure S12**: Biological sampling effort in Brazil. Red indicates areas with high sampling effort. *Map created in Dinamica EGO (https://csr.ufmg.br/dinamica/).*
